# Association of composite dietary antioxidant index with circadian syndrome: evidence from NHANES

**DOI:** 10.3389/fnut.2024.1501352

**Published:** 2025-01-03

**Authors:** Chen Chen, Chenyu Zhao, Hongyu Jin, Zhiping Jiang, Wei Wang, Wen-Yang Li

**Affiliations:** ^1^Respiratory and Critical Care Department, The First Hospital of China Medical University, Shenyang, China; ^2^Department of China Medical University, School of Pharmacy, The Queen’s University of Belfast Joint College, China Medical University, Shenyang, China

**Keywords:** composite dietary antioxidant index, dietary antioxidant, circadian syndrome, cross-sectional study, NHANES, restricted cubic spline

## Abstract

**Background:**

The Circadian Syndrome (CircS) has been linked to various chronic diseases. However, the relationship between composite dietary antioxidant index (CDAI) and CircS has remained unexplored. This study aimed to investigate the potential association between CDAI and CircS.

**Methods:**

Cross-sectional analyses were based on the National Health and Nutrition Examination Survey (NHANES) 2005–2018. Dietary consumption was assessed via the 24-h diet recall method and CDAI was computed following a validated approach involving six antioxidants. CircS was defined based on metabolic syndrome components, supplemented by short sleep duration and depressive symptoms. The relationship between CDAI and CircS was examined using weighted multivariable logistic regression and subgroup analyses. Additionally, restricted cubic spline (RCS) regression was employed to investigate potential nonlinear correlations.

**Results:**

Among 11,048 subjects included (mean age 47.57 years), 2,733 (weighted prevalence = 22.13%) were reported to have CircS. Logistic regression revealed that the highest quartile of CDAI was inversely associated with the risk of CircS {odds ratio (OR) [95% CI = 0.69 (0.55–0.87)]} and the risk of depression [OR = 0.59 (0.48–0.72)], short sleep duration [OR = 0.54 (0.41–0.70)], elevated fasting glucose [OR = 0.80 (0.65–0.98)], elevated triglycerides (TG) [OR = 0.74 (0.59–0.92)], elevated waist circumference [OR = 0.65, (0.52–0.80)] and reduced high-density lipoprotein cholesterol (HDL-C) [OR = 0.75 (0.61–0.92)], respectively. A dose–response gradient in odds of CircS components was noted as CDAI levels increased, particularly with depression and short sleep duration. RCS showed a non-linear relationship between CDAI and CircS, with a U-shaped correlation found between Zinc and CircS (inflection point 12.63). Subgroup analysis showed BMI modified the inverse association between CDAI and CircS (*p* for interaction = 0.003).

**Conclusion:**

This study revealed a non-linear and negative association between CDAI and CircS risk, with a U-shaped correlation observed between Zinc and CircS. Obese individuals might not benefit from excessively high CDAI. The results suggest that a higher CDAI score was correlated with a decreased risk of CircS.

## 1 Introduction

Circadian rhythms arise as a biological response to Earth’s rotation, encompass physical, mental, and behavioral changes following a 24-h cycle (1). Both circadian rhythms and oxygen play essential roles in maintaining homeostasis through various physiological processes (2). Previous clinical guidelines did not attribute a unified etiological characteristic to metabolic syndrome (METS), rendering it a vague concept that overlooks common comorbidities like depression and sleep disturbance (3). The newly defined circadian syndrome (CircS) is an innovative concept stemmed from metabolic syndrome but now encompasses additional comorbidities such as short sleep duration, and depression (4, 5). Circadian rhythm disruptions have become increasingly common in recent years, attributed to factors such as exposure to artificial lighting at night, irregular meal timing, and occupations involving shift or night work (6, 7). CircS has been introduced to collectively address a cluster of cardiometabolic risk factors that increases susceptibility to cardiovascular disease (CVD) and type 2 diabetes mellitus (4, 8). Given the crucial role of CircS in predicting cardiovascular disease (CVD) and type 2 diabetes mellitus (T2DM) (9), understanding its root causes and identifying modifiable risk factors are imperative for developing effective prevention and management strategies for cardio-metabolic conditions (9, 10).

Oxidative stress is a key factor in many diseases, it disrupts the balance between antioxidants and oxidants in the body through the generation of reactive substances and redox signaling (11). Environmental stimuli, as well as prolonged unhealthy dietary and lifestyle habits, can trigger oxidative stress in cells (12). Consuming sufficient dietary antioxidants can maintain the body’s redox balance, thus averting oxidative stress (13–15). The Composite Dietary Antioxidant Index (CDAI) serves as a valuable nutritional metric that assesses an individual’s antioxidant status by considering the intake of diverse dietary vitamins and minerals possessing antioxidant properties from daily food and beverage consumption. This index typically includes vitamins A, C, E, selenium, zinc, and carotenoids in its evaluation (16, 17). CDAI was employed to explore the correlation between dietary antioxidant capacity and chronic conditions such as cardiovascular disease, cancer, and diabetes, with elevated CDAI values linked to the enhanced health outcomes (18–21). Lipid peroxidation, protein oxidation, and DNA damage caused by reactive oxygen species (ROS) may exhibit diurnal variations (22, 23). Therefore, it is reasonable to suggest that CircS could be linked to the circadian fluctuation of oxidative stress reactions. Utilizing the CDAI may offer insights into the complex relationship between dietary antioxidants, CircS and human health, potentially leading to more tailored and individualized dietary suggestions. Diets rich in antioxidants may play a crucial role in reducing the risk of CircS (24–26).

.Recent studies revealed that poor dietary patterns raise the risk of CircS, and pro-oxidation diets are also related with metabolic disorders, emphasizing the importance of healthy eating habits in lowering the risk of Circs (9, 21). This study aims to utilize NHANES public database to explore the potential relationship between CDAI and CircS in American adults. We hypothesize that higher CDAI levels are associated with a reduced risk of CircS, possibly due to the antioxidant properties of vitamins and minerals, which in turn mitigate oxidative stress.

## 2 Methods

### 2.1 Study design and population

This prospective cohort study used a nationally representative sample from the US National Health and Nutrition Examination Survey (NHANES), which has been conducted on 2-year cycles since 1999 to monitor the health and nutritional status of the US population (27). All the NHANES protocols were approved by the National Center for Health Statistics ethics review board, and written informed consent was obtained from all participants (28). This modeling investigation was exempt from review as it used to publish deidentified data sets that included no personally identifiable information.^1^

**Figure 1:**
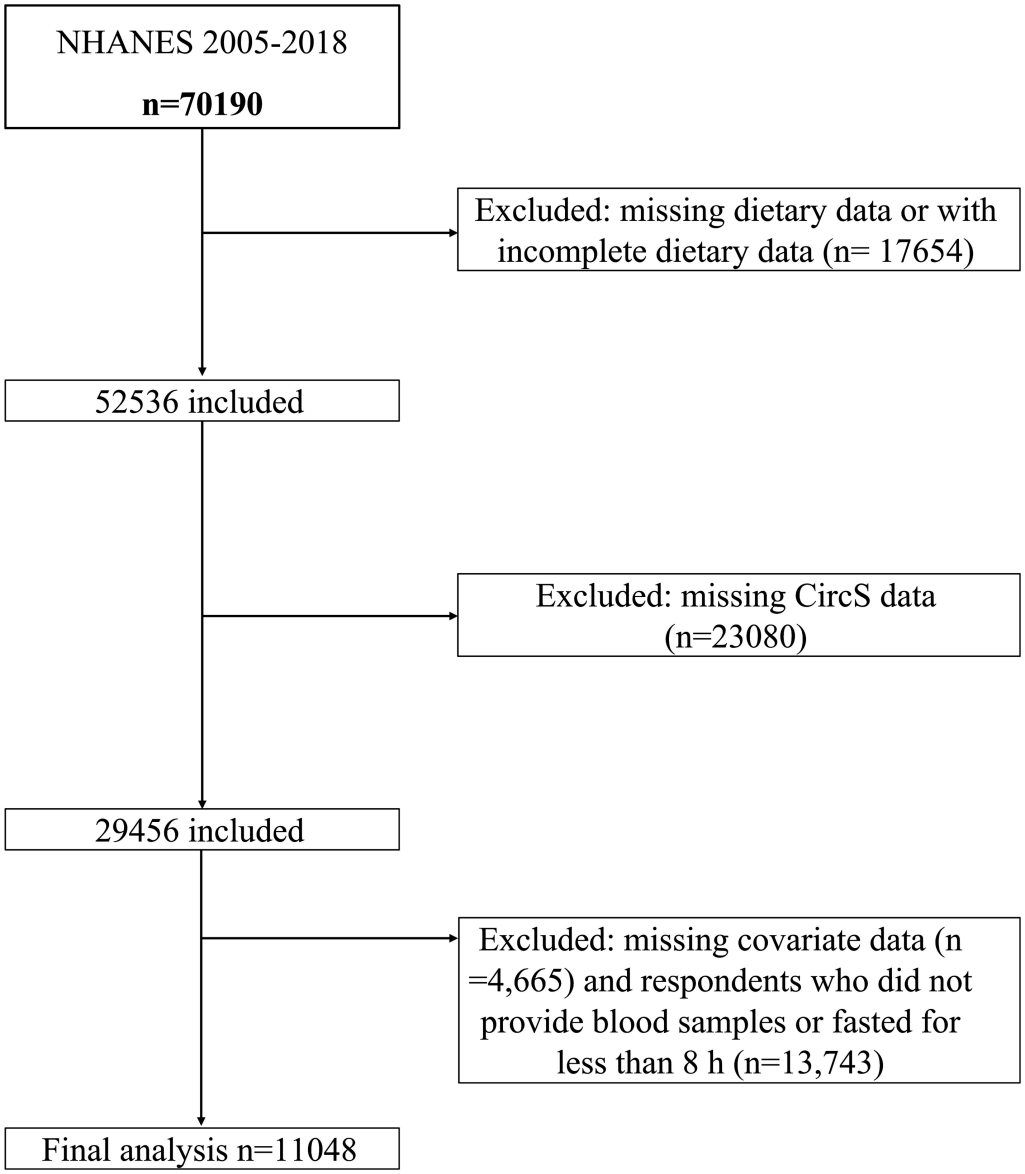
Flowchart of the study design.

The study utilized data from seven consecutive survey cycles of NHANES spanning from 2005 to 2018, involving 70,190 participants. Exclusion criteria consisted of: (1) missing or incomplete dietary data (e.g., participants with only 1 day of dietary records instead of two, and those lacking dietary interview data, *n* = 17,654); (2) absence of CircS data (*n* = 23,080); (3) missing covariate data (*n* = 4,671); and (4) individuals who did not provide blood samples or fasted for less than 8 h (*n* = 13,740) were excluded. Ultimately, the study included 11,048 eligible participants. The participant inclusion flowchart is depicted in Figure 1.

### 2.2 Assessment of composite dietary antioxidant index

The information on dietary and their components was collected by highly trained staffs in NHANES. NHANES collected food intake data via nonconsecutive 24-h dietary recall interviews spanning 2 days. The initial dietary recall interview took place in person at the Mobile Examination Center (MEC), with the subsequent interview conducted over the phone 3 to 10 days later. Daily average intakes were computed from the two-day dietary recall data for the final statistical analysis to mitigate bias. The calculation of CDAI was derived from six antioxidant components: vitamin A, vitamin C, vitamin E, zinc, selenium, and carotenoids. CDAI was calculated as the total of daily average intakes of vitamins A, C, and E, zinc, selenium, and carotenoids, which were normalized by subtracting the mean and dividing by their standard deviation (SD) (29).


$$\mathrm{CDAI}=\overset{n-a6}{\underset{i=1}{\sum }}\left(\mathrm{Individual}\;\mathrm{Intake}-\mathrm{Mean}\right)/SD)$$

### 2.3 Assessment of circadian syndrome

MetS was defined based on the harmonized criteria outlined in the joint interim statement by the International Diabetes Federation Task Force on Epidemiology and Prevention, National Heart, Lung, and Blood Institute, American Heart Association, World Heart Federation, International Atherosclerosis Society, and International Association for the Study of Obesity (30). METS was defined as the presence of 3 or more of the following components, These include (1) elevated waist circumference (WC): ≥102 cm in males and ≥ 88 cm in females; (2) elevated fasting glucose: ≥ 100 mg/dL or drug treatment; (3) elevated triglycerides (TG): serum TG ≥ 150 mg/dL (1.7 mmol/L) or drug treatment; (4) reduced high density lipoprotein cholesterol (HDL-C): serum HDL-C < 40 mg/dL (1.03 mmol/L) in men and < 50 mg/dL (1.29 mmol/L) in women or specific treatment; (5) elevated blood pressure: systolic BP ≥130 mmHg or diastolic BP ≥ 85 mmHg or antihypertensive drug use in patients with hypertension.

CircS assessment included seven components: self-reported short sleep (<6 h/day), depressive symptoms, and the five components utilized in defining MetS (31). Participants with a PHQ-9 score of 5 were defined as having depression symptoms. Based on MetS and CircS, a third variable was constructed for each individual with a possible value of: normal, MetS alone, CircS alone, and both MetS and CircS (32).

### 2.4 Covariates

Our aim was to mitigate potential confounding biases in our analysis by selecting covariates grounded in prior research and clinical plausibility. The selected covariates comprised age (continuous or categorical: <40, 40–59, or ≥ 60 years), sex (male or female), race (Non-Hispanic White, Non-Hispanic Black, and other races), poverty income ratio (PIR, classified as <1, and ≥ 1) educational level (less than high school, and high school or above), and body mass index (BMI) classified as <30 kg/m^2^, and ≥ 29.9 kg/m^2^, representing underweight/normal, overweight, and obesity, respectively. Smoking status was classified as non-smokers, former smokers, or current smokers, while alcohol consumption was denoted by yes or no. Additionally, total energy intake (kcal) and dietary cholesterol intake (mg/day), assessed through two 24-h recalls, were taken into account in our study. Physical activity (measured as Metabolic Equivalent of Task (METs) minutes per week and recoded into <600, 600–1,200, and 1,200 MET min/week). These variables were incorporated as covariates to address their potential impact on the outcomes of interest.

### 2.5 Statistical analysis

Data analysis in this study followed the analytical guidelines set by the National Center for Health Statistics (NCHS). The data were merged and weighted using WTSFA2YR according to NHANES recommendations. Baseline characteristics were compared between participants with and without CircS using the weighted Student’s t-test and the chi-square test. Categorical variables are presented as numbers (weighted percentages), and continuous variables are given as weighted means (standard errors). CDAI scores were evenly divided into four quartiles, with the first quartile (Q1) as the reference point. Multivariable logistic regression analysis was applied to identify the relationship between CDAI (and its components) and CircS (and its components). In the crude model, no covariates were adjusted; in model 1, age, sex (male/female), race (Non-Hispanic White, Non-Hispanic Black, and other races), educational level (less than high school, and high school or above), and energy intake (kcal/day) were adjusted; model 2 was additionally adjusted for cholesterol intake (mg/d), smoke status (non-smokers, former-smokers, or current smokers), and alcohol consumption (yes/no). Moreover, a restricted cubic spline (RCS) analysis was performed to explore the nonlinear relationship between CDAI (and its components) and CircS (and its components). Subgroup analyses were performed to determine whether the risk for CircS in relation to CDAI according to age, sex, race, educational level, obesity status, smoking status, and alcohol consumption. In order to assess the robustness of our findings, a series of sensitivity analyses were performed. This included unweighted logistic regression; furthermore, we reanalyzed the regression models using data spanning 2007–2018, incorporating physical activity as supplementary covariates. All analyses were performed using R.^2^ *p* < 0.05 was regarded as statistically significant.

## 3 Results

### 3.1 Baseline characteristics of the participants

Table 1 displays the initial NHANES characteristics for adult individuals from 2005 to 2018. The analysis included 11,048 adults, of whom 2,733 had CircS. The sample size from NHANES 2005–2018 was 11,048, representing a total population-based size of 163,381,685 individuals in the United States. The average age of the entire study population was 47.57 years, with females comprising 51.60%. Individuals with CircS tended to be older, female, non-Hispanic white, less educated, and obese. The average CDAI score in the study population was 0.96. Individuals with CircS had lower CDAI scores compared to those without (0.61 vs. 1.06). Supplementary Table S1 presented the demographic and clinical attributes of the study population categorized by CDAI quartiles. The score range was ≤ −2.191 in Q1, −2.191 < CDAI ≤0.044 in Q2, 0.046 < CDAI ≤2.765 in Q3, and > 2.765 in Q4. With increasing quartiles, a decreasing proportion of participants were diagnosed with CircS. The weighted prevalence of normal individuals was 64.63%, 146 (1.09%) had CircS alone, 1,514 (13.24%) had MetS alone, and 2,587 (21.04%) had both CircS and MetS (Supplementary Table S2).

**Table 1:** Baseline characteristics by Circadian syndrome status: NHANES 2007 -2012.

Characteristic	Total	No circadian syndrome	Circadian syndrome	*p* value
	*n* = 11,048	*n* = 8,315	*n* = 2,733	
Age, years	47.57 (0.28)	45.54 (0.31)	54.74 (0.35)	<0.001
Energy intake, kcal	4206.59 (20.34)	4243.40 (21.77)	4077.08 (39.84)	<0.001
Cholesterol intake, mg/d	286.50 (2.38)	285.73 (2.78)	289.20 (4.57)	0.520
Vitamin A, mcg	644.96 (8.42)	652.33 (10.24)	619.01 (16.08)	0.100
Vitamin C, mg	80.28 (1.22)	82.06 (1.39)	74.01 (1.65)	<0.001
Vitamin E, mg	8.43 (0.08)	8.60 (0.09)	7.81 (0.13)	<0.001
Zinc, mg	11.57 (0.09)	11.59 (0.08)	11.50 (0.24)	0.700
Selenium, mcg	114.25 (0.67)	115.08 (0.70)	111.34 (1.23)	0.004
Carotenoid, mcg	9964.71 (178.27)	10164.96 (207.03)	9260.20 (254.31)	0.004
CDAIQ				<0.001
Q1	2,763 (21.68)	1965 (20.66)	798 (25.25)	
Q2	2,761 (24.40)	2056 (24.28)	705 (24.84)	
Q3	2,762 (26.44)	2,145 (27.00)	617 (24.49)	
Q4	2,762 (27.48)	2,149 (28.06)	613 (25.41)	
Age group, *n* (%)				<0.001
<40	3,522 (35.25)	3,098 (40.50)	424 (16.79)	
40–59	3,791 (38.63)	2,780 (37.25)	1,011 (43.52)	
≥60	3,735 (26.11)	2,437 (22.25)	1,298 (39.69)	
Sex, *n* (%)				<0.001
Male	5,400 (48.40)	4,254 (49.86)	1,146 (43.25)	
Female	5,648 (51.60)	4,061 (50.14)	1,587 (56.75)	
Race, *n* (%)				0.280
Non-Hispanic Black	3,703 (18.78)	2,847 (19.12)	856 (17.61)	
Non-Hispanic White	2,166 (10.37)	1,642 (10.29)	524 (10.66)	
Other	5,179 (70.85)	3,826 (70.60)	1,353 (71.73)	
BMI, *n* (%)				<0.001
<30.0 kg/m2	6,782 (62.61)	5,886 (71.64)	896 (30.85)	
≥30.0 kg/m2	4,266 (37.39)	2,429 (28.36)	1837 (69.15)	
Educational level, *n* (%)				<0.001
High school or above	8,674 (85.92)	6,708 (87.48)	1966 (80.43)	
Less than high school	2,374 (14.08)	1,607 (12.52)	767 (19.57)	
Marital status, *n* (%)				0.080
Married	6,792 (65.19)	5,176 (65.79)	1,616 (63.08)	
Other	4,256 (34.81)	3,139 (34.21)	1,117 (36.92)	
Family PIR, *n* (%)				<0.001
<1	2072 (12.42)	1,456 (11.72)	616 (14.89)	
≥1	8,976 (87.58)	6,859 (88.28)	2,117 (85.11)	
Smoking status, *n* (%)				<0.001
Never	6,097 (55.04)	4,790 (57.49)	1,307(46.44)	
Former	2,815 (25.91)	1997 (24.33)	818 (31.47)	
Current	2,136 (19.05)	1,528 (18.18)	608 (22.09)	
Alcohol consumption, *n* (%)				0.010
No	1,407 (10.19)	1,017 (9.82)	390 (11.47)	
Yes	9,641 (89.81)	7,298 (90.18)	2,343 (88.53)	

Continuous variables were showed as mean (SE), categorical variables were showed as *n* (%); All estimates accounted for complex survey designs, and all percentages were weighted; *n*, sample size; SE, standard error; Q: quartile; BMI, body mass index; CDAI, composite dietary antioxidant index; Q1: CDAI ≤ −2.191; Q2: -2.191 < CDAI ≤ 0.044; Q3: 0.046 < CDAI ≤ 2.765; Q4: CDAI > 2.765.

### 3.2 Association between CDAI and CircS

**Figure 2:**
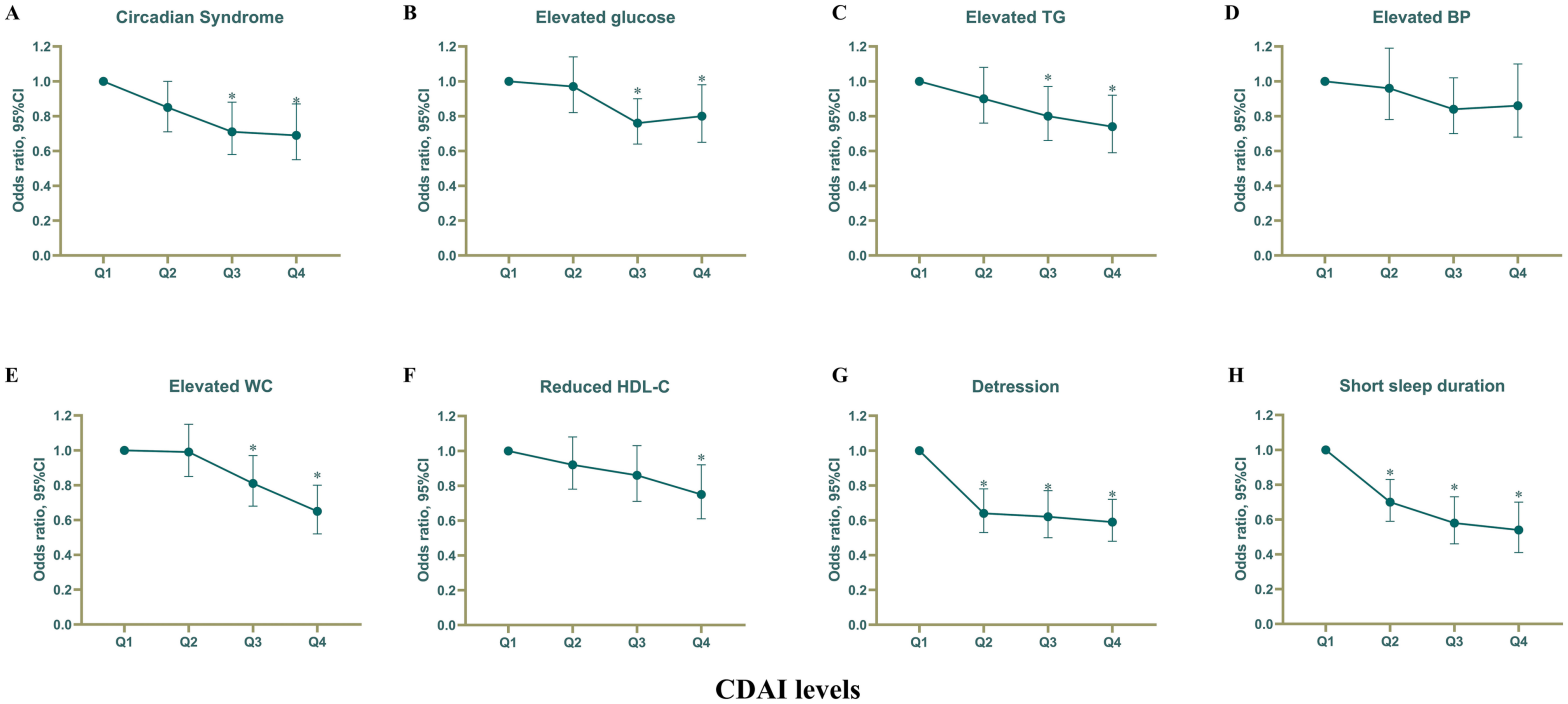
Association of CDAI with Circs and its components. Associations of CDAI with **(A)** Circadian Syndrome, **(B)** elevated glucose, **(C)** elevated TG, **(D)** elevated BP, **(E)** elevated WC, **(F)** reduced HDL-C, **(G)** depression, and **(H)** Short sleep duration. CDAI, Composite dietary antioxidant index; Circs, Circadian Syndrome. *Statistically significant findings. Adjusted for age, sex, race, poverty income ratio, educational level, energy intake, cholesterol intake, smoke status, and alcohol consumption.

We discovered a significant inverse relationship between categorical CDAI (divided into quartiles), CircS, and its six components (all *p*-values for trend<0.05). In the fully adjusted model 2, elevated CDAI was linked to decreased odds of CircS [Q2 vs. Q1: odds ratio (OR) = 0.85, 95% confidence interval (CI) 0.71–1.00, *p* = 0.053; Q3 vs. Q1: OR = 0.71 (0.58–0.88), *p* = 0.001; Q4 vs. Q1: OR = 0.69 (0.55–0.87), *p* = 0.001] (Figure 2A; Table 2). Compared to the first quartile, the fourth quartile of CDAI was found to have a negative association with an increased risk of elevated fasting glucose [OR = 0.80 (0.65–0.98), *p* for trend = 0.7], elevated TG [OR = 0.74 (0.59–0.92), *p* for trend = 0.005], elevated waist circumference [OR = 0.65 (0.52–0.80), *p* for trend<0.001], reduced LDL-C [OR = 0.75 (0.61–0.92), *p* for trend = 0.006], depression [OR = 0.59 (0.48–0.72), *p* for trend<0.001], and short sleep duration [OR = 0.54 (0.41–0.70), *p* for trend<0.001]. (Figure 2; Supplementary Table S3). Nevertheless, there was no observed association between CDAI and elevated blood pressure. The most significant decrease in odds was observed for short sleep when transitioning from Q1 to Q4, showing a 46% reduction. Depression experienced the second-largest decrease in odds, with a 41% decrease (Figure 2; Supplementary Table S3). There were modest variations in odds among other components when comparing high CDAI with low CDAI. A negative correlation was noted between per 1-SD increase in CDAI and CircS along with its components (all *p*-values <0.05 except for elevated fasting glucose).

**Table 2:** Weighted logistic regression analysis on the association between CDAI and CircS.

	Crude model		Model 1		Model 2	
	OR 95% CI	*p*	OR 95% CI	*p*	OR 95% CI	*p*
Circs						
Q1	1 [Reference]		1 [Reference]		1 [Reference]	
Q2	0.84 (0.72,0.98)	0.024	0.81 (0.69,0.97)	0.012	0.85 (0.71,1.00)	0.053
Q3	0.74 (0.62,0.89)	0.002	0.69 (0.56,0.85)	<0.001	0.71 (0.58,0.88)	0.001
Q4	0.74 (0.63,0.87)	<0.001	0.65 (0.52,0.82)	<0.001	0.69 (0.55,0.87)	0.001
*p* for trend		<0.001		<0.001		<0.001
Per 1-SD increase	0.89 (0.83,0.95)	<0.001	0.83 (0.75,0.92)	<0.001	0.85 (0.76,0.94)	0.003

Crude model: Unadjusted model. Model 1: Adjusted for age, sex, race, PIR, educational level and energy intake. Model 2: Additionally adjusted for cholesterol intake, smoke status, and alcohol consumption. CDAI, composite dietary antioxidant index; CircS, circadian syndrome; PIR, poverty income ratio; SD, standard deviation.

**Figure 3:**
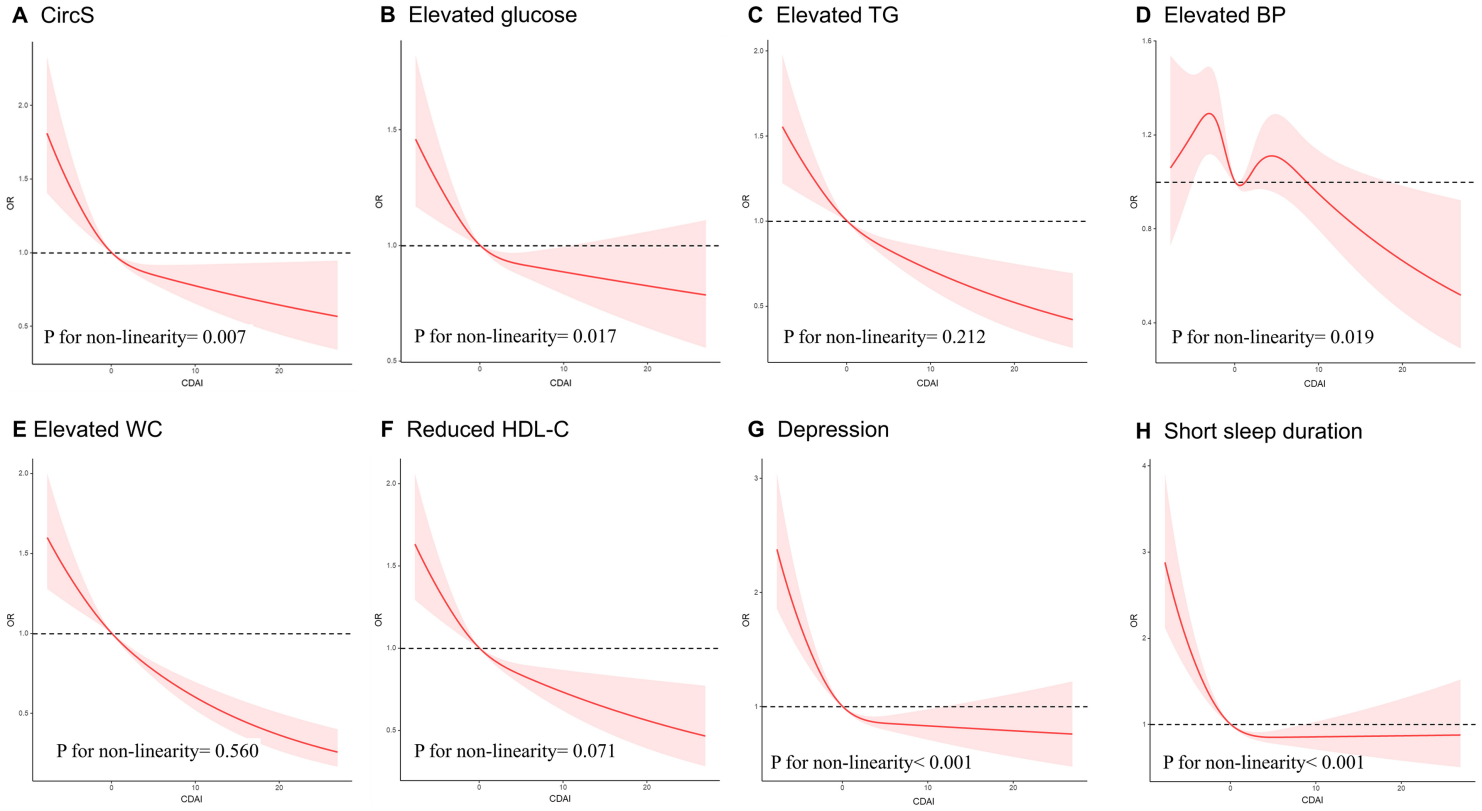
The RCS curve of the association of CDAI with CircS and its components among all the study participants. **(A)** The RCS curve of the association between CDAI and CircS; **(B)** The RCS curve of the association between CDAI and elevated glucose; **(C)** The RCS curve of the association between CDAI and elevated TG; **(D)** The RCS curve of the association between CDAI and elevated BP; **(E)** The RCS curve of the association between CDAI and elevated WC; **(F)** The RCS curve of the association between CDAI and reduced HDL-C; **(G)** The RCS curve of the association between CDAI and depression. **(H)** The RCS curve of the association between CDAI and short sleep duration. RCS regression was adjusted for age, sex, race, PIR, educational level, energy intake, cholesterol intake, smoke status, and alcohol consumption. RCS, restricted cubic spline; CDAI, composite dietary antioxidant index; CircS, circadian syndrome; TG, triglycerides; BP, blood pressure; WC, waist circumference; HDL-C, High density lipoprotein-cholesterol, PIR, poverty income ratio.

Figure 3 displays the analysis of the RCS regression. After adjusting for all covariates, a notable nonlinear correlation was observed between CDAI and CircS (*p* for nonlinearity = 0.007). As CDAI scores increased, the likelihood of CircS showed a gradual decrease until reaching a specific threshold, after which the protective effect appeared to stabilize, indicating a potential saturation effect at higher CDAI values. Similarly, a non-linear association was observed between CDAI and the individual components of CircS (*p* for nonlinearity <0.05), except for elevated TG and waist circumference.

### 3.3 Individual CDAI components and CircS

**Figure 4:**
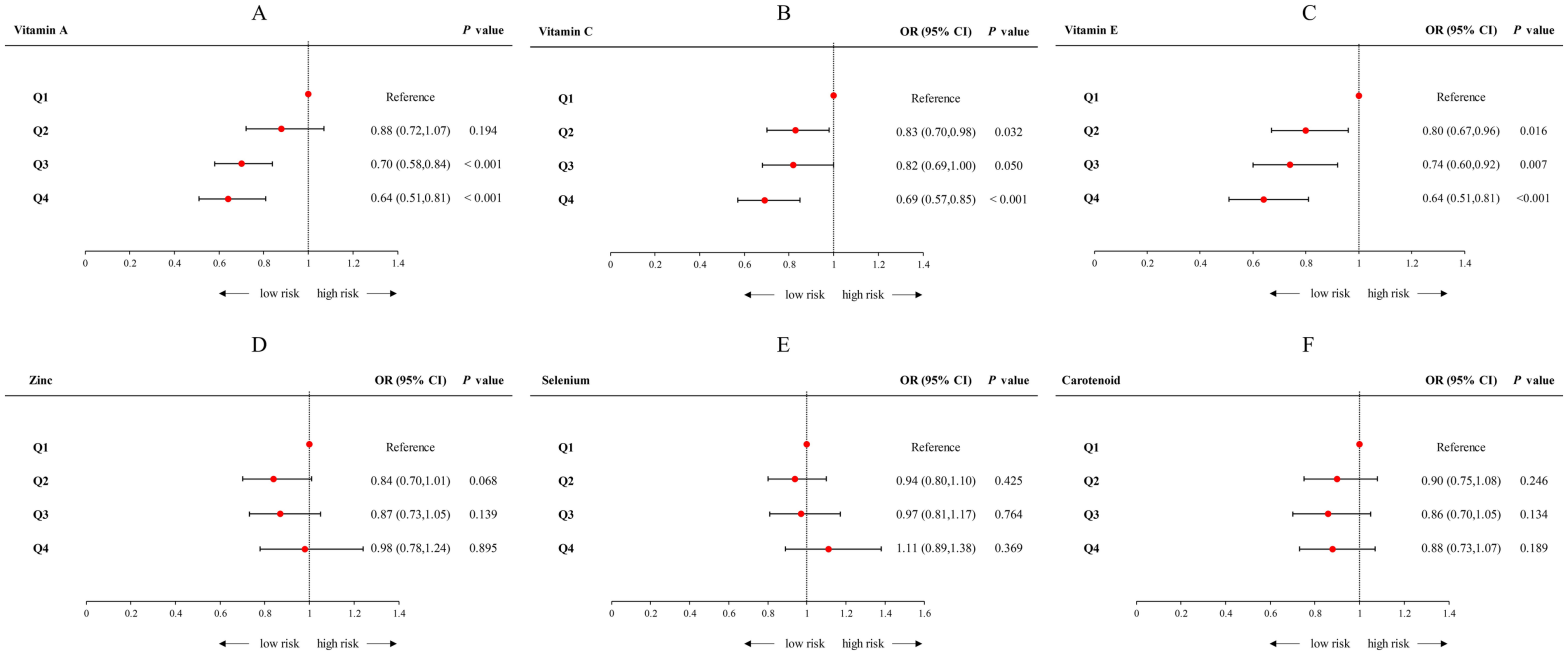
Forest plot of logistic regression analyses of the association between CDAI components and Circs. **(A)** The logistic regression analyses of the association between Vitamin A and Circs; **(B)** The logistic regression analyses of the association between Vitamin C and Circs; **(C)** The logistic regression analyses of the association between Vitamin E and Circs; **(D)** The logistic regression analyses of the association between Zinc and Circs; **(E)** The logistic regression analyses of the association between Selenium and Circs; **(F)** The logistic regression analyses of the association between Carotenoid and Circs. Adjusted for age, sex, race, PIR, educational level, energy intake, cholesterol intake, smoke status, and alcohol consumption. CDAI, composite dietary antioxidant index; CircS, circadian syndrome; PIR, poverty income ratio.

The correlation between specific antioxidant components and CircS was examined by categorizing individual antioxidants into quartiles, with the first quartile (Q1) as the reference group (Figure 4; Supplementary Table S4). After adjusting all confounding factors, the OR (95% CI) when comparing the highest quartile (Q4) with the first quartile (Q1) was 0.64 (0.51–0.81), *p* for trend <0.001 for vitamin A; 0.69 (0.57–0.85), *p* for trend <0.001 for vitamin C; and 0.64 (0.51–0.81), *p* for trend <0.001 for vitamin E, respectively. Conversely, regarding other antioxidants, these inverse relationships were not observed in the cases of Zinc,selenium, and carotenoids in logistic regression.

**Figure 5:**
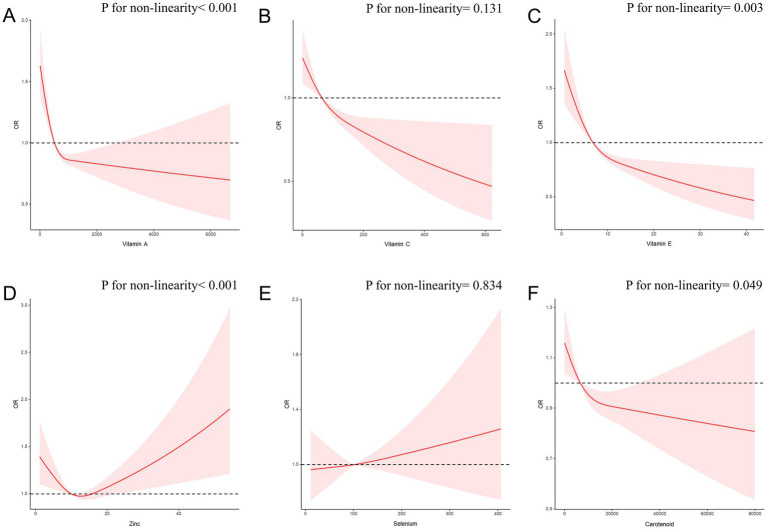
The RCS curves of the association between CDAI components and CircS among all the study participants. **(A)** The RCS curve of the association between Vitamin A and circadian syndrome; **(B)** The RCS curve of the association between Vitamin C and circadian syndrome; **(C)** The RCS curve of the association between Vitamin E and circadian syndrome; **(D)** The RCS curve of the association between zinc and circadian syndrome; **(E)** The RCS curve of the association between selenium and circadian syndrome; **(F)** The RCS curve of the association between carotenoid and circadian syndrome. RCS regression was adjusted for age, sex, race, PIR, educational level, energy intake, cholesterol intake, smoke status, and alcohol consumption. RCS, restricted cubic spline; CDAI, composite dietary antioxidant index; CircS, circadian syndrome; PIR, poverty income ratio.

Furthermore, we investigated the associations of each individual component of the CDAI with CircS using RCS analysis (Figure 5). Notably, vitamin A, vitamin E, and carotenoids all exhibited a non-linear negative correlation with CircS (*p* for non-linearity<0.05). Zinc intake demonstrated a nonlinear U-shaped dose–response association, with an inflection point at 12.63 (*p* for non-linearity<0.001). However, we found no evidence of a non-linear relationship between vitamin C intake (*p* for non-linearity = 0.131), selenium intake (*p* for non-linearity = 0.834) and CircS.

### 3.4 Subgroup and sensitivity analysis

The study population was analyzed in subgroups based on age, sex, race, poverty income ratio, educational level, obesity status, smoking status, and alcohol consumption (Table 3). Significant interactions were observed between CDAI and BMI status in relation to CircS (*p* for interaction =0.003). Notably, higher CDAI levels may provide more benefits for non-obese individuals, whereas no significant relationship was observed for obese individuals.

**Table 3:** Subgroups analyses of the associations between quartiles of CDAI and CircS.

	Quartiles of composite dietary antioxidant index					
	Quartile 1	Quartile 2	Quartile 3	Quartile 4	*p* for trend	*p* for interaction
Age group						0.799
<40	1 [Reference]	0.99 (0.72,1.37)	0.77 (0.50,1.16)	0.64 (0.42,0.97)	0.020	
40–59	1 [Reference]	0.84 (0.62,1.14)	0.66 (0.48,0.90)	0.69 (0.49,0.97)	0.030	
≥60	1 [Reference]	0.82 (0.63,1.07)	0.81 (0.59,1.11)	0.78 (0.56,1.08)	0.224	
Sex						0.645
Male	1 [Reference]	0.88 (0.67,1.16)	0.73 (0.54,0.98)	0.75 (0.54,1.06)	0.109	
Female	1 [Reference]	0.80 (0.64,0.99)	0.68 (0.51,0.91)	0.63 (0.47,0.85)	0.005	
Race						0.44
Non-Hispanic Black	1 [Reference]	0.92 (0.69,1.24)	0.74 (0.52,1.05)	0.73 (0.49,1.09)	0.124	
Non-Hispanic White	1 [Reference]	1.14 (0.84,1.53)	0.81 (0.56,1.18)	0.98 (0.59,1.64)	0.681	
Other	1 [Reference]	0.78 (0.62,0.99)	0.69 (0.52,0.90)	0.65 (0.48,0.88)	0.010	
PIR						0.312
<1	1 [Reference]	1.07 (0.80,1.43)	0.72 (0.49,1.05)	0.93 (0.60,1.43)	0.445	
≥1	1 [Reference]	0.81 (0.66,0.98)	0.70 (0.55,0.89)	0.66 (0.51,0.84)	0.003	
Educational level						0.207
Less than high school	1 [Reference]	0.80 (0.66,0.98)	0.69 (0.55,0.87)	0.64 (0.50,0.83)	0.002	
High school or above	1 [Reference]	1.01 (0.74,1.38)	0.73 (0.49,1.08)	1.02 (0.68,1.54)	0.728	
BMI						0.003
<30.0 kg/m2	1 [Reference]	0.67 (0.51,0.90)	0.68 (0.50,0.92)	0.51 (0.36,0.73)	<0.001	
≥30.0 kg/m2	1 [Reference]	0.92 (0.72,1.16)	0.77 (0.58,1.03)	0.97 (0.70,1.33)	0.874	
Smoking status						0.260
Never	1 [Reference]	0.92 (0.72,1.17)	0.72 (0.55,0.94)	0.61 (0.47,0.80)	<0.001	
Former	1 [Reference]	0.60 (0.45,0.81)	0.53 (0.39,0.74)	0.58 (0.38,0.90)	0.084	
Current	1 [Reference]	1.04 (0.74,1.48)	0.99 (0.61,1.59)	1.22 (0.74,2.01)	0.532	
Alcohol consumption						0.433
No	1 [Reference]	0.81 (0.50,1.31)	0.67 (0.38,1.15)	0.86 (0.49,1.49)	0.635	
Yes	1 [Reference]	0.84 (0.70,1.02)	0.71 (0.57,0.90)	0.67 (0.52,0.87)	0.003	

Analyses were adjusted for age, sex, race, PIR status, educational level, energy intake, cholesterol intake, smoke status, and alcohol consumption when they were not the strata variables. CDAI, composite dietary antioxidant index; CircS, circadian syndrome; PIR, poverty income ratio.

**Figure 6:**
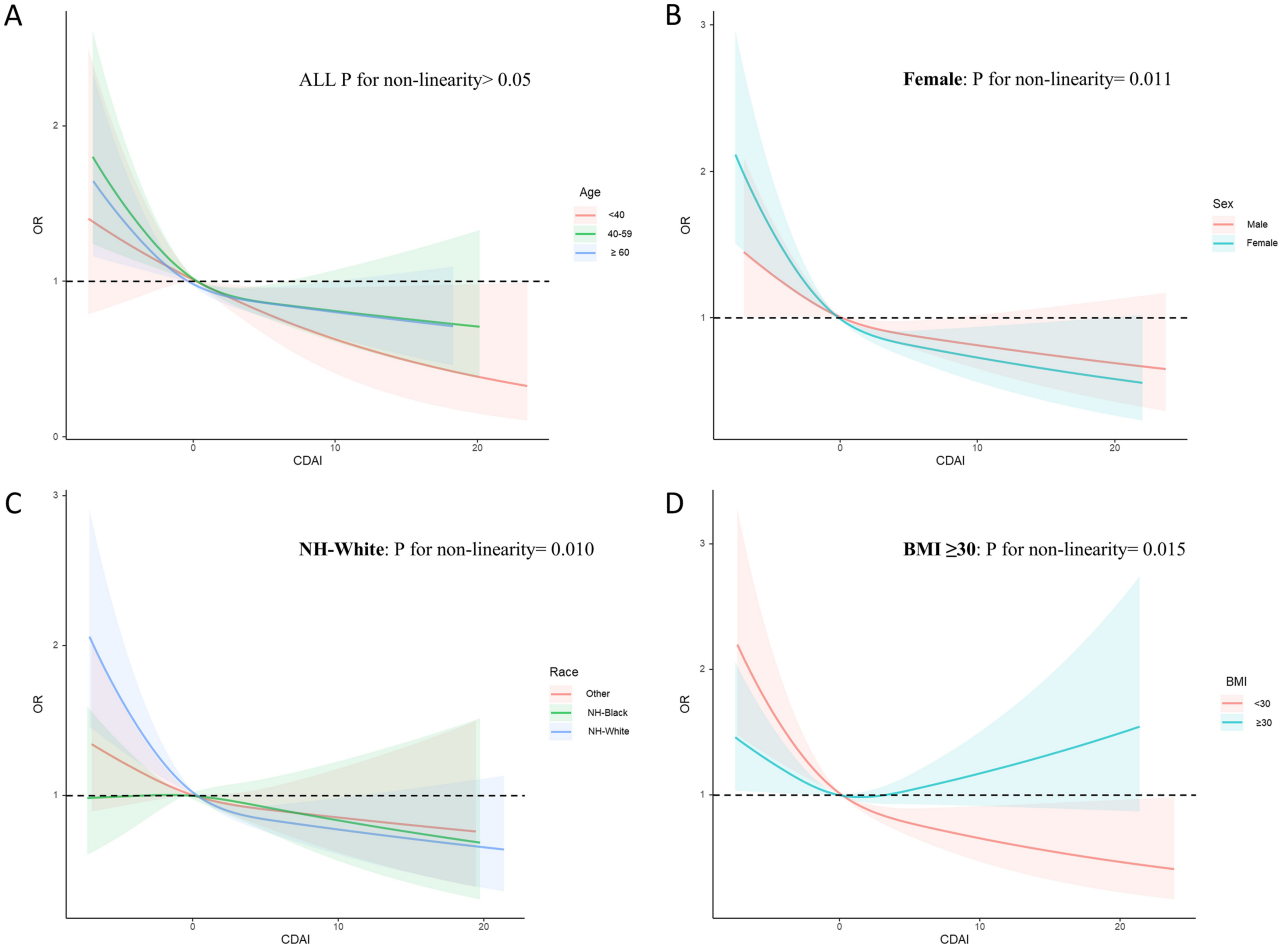
Subgroups RCS analyses for the association between CDAI and circadian syndrome stratified by **(A)** age (<40, 40–59, and ≥ 60), **(B)** sex (male and female), **(C)** race (others; NH-Black, and NH-White), and **(D)** BMI (<30.0, and ≥ 30.0). RCS regression was adjusted for age, sex, race, PIR, educational level, energy intake, cholesterol intake, smoke status, and alcohol consumption. RCS, restricted cubic spline; CDAI, composite dietary antioxidant index. CircS, circadian syndrome, PIR, poverty income ratio.

Additionally, subgroup RCS analysis revealed that increasing CDAI levels might result in greater benefits for participants aged 40–59 years, females, non-Hispanic White individuals, and both obese and non-obese participants (Figure 6). Among non-obese participants, the risk of CircS decreased as CDAI increased. In contrast, for obese participants, a U-shaped non-linear negative correlation was identified between CDAI and CircS (*p* for nonlinearity = 0.015) with an inflection point at 1.55.

To enhance the validation of the results in this study, we conducted a series of sensitivity analyses, including using unweighted logistic regression (Supplementary Table S5) and adjusted for physical activity as supplementary covariates using data from 2007 to 2018 (Supplementary Table S6 and Supplementary Figure S1). The results remained generally robust in the sensitivity analyses, showing that higher CDAI scores continued to exhibit a positive correlation with a reduced risk of CircS.

## 4 Discussion

To the best of our knowledge, this study represented the first large-sample investigation into the association between CDAI and CircS risk. Although dietary antioxidants have been a key focus in metabolic syndrome prevention, existing research on this relationship was relatively sparse (33). Our findings indicated that higher CDAI scores were associated with a decreased risk of CircS, with depression and short sleep duration being more prominent. A U-shape correlation was found between Zinc and CircS. Obese individuals might not have derived benefits from excessively high CDAI values.

Two additional components, depression and short sleep duration, were added to MetS to form CircS, which now requires 4 components instead of 3. Without this refinement, 2,587 participants (weighted prevalence = 21.04%) initially classified with CircS would have been misclassified with MetS. With this updated definition, 1,514 participants (13.24%) identified with MetS alone did not meet CircS criteria. Despite the stricter criteria, 146 participants (1.09%) classified as CircS alone did not have MetS. This highlights the added value of including depression and short sleep in CircS definition. Wang et al. (34) analyzed NHANES data from 2005 to 2018 and linked CircS with stroke. Studies in Chinese and American populations have shown CircS exhibited superior predictive value for CVD and cognitive function compared to MetS (5, 31, 32)

The emerging circadian syndrome (CircS) encompassed key metabolic syndrome elements and related comorbidities such as sleep disturbance and depression, drawing increased attention (4). Understanding CircS root causes and identifying modifiable risk factors are crucial for effective prevention and management. ROS play a pivotal role in cardiometabolic disease pathogenesis, and dietary antioxidants can mitigate ROS levels (35). In this study, dietary habits were incorporated to comprehensively evaluate the potential relationship between oxidative stress and CircS. According to our investigation, negative correlations were elucidated between CDAI and various CircS components, including elevated waist circumference, elevated fasting glucose, elevated TG, reduced HDL-C, depression symptoms, and short sleep duration, except for elevated blood pressure. International studies have shown conflicting results regarding the relationship between dietary antioxidant capacity (DTAC) and CircS components. For instance, a Korean study demonstrated a negative association between DTAC and serum TG levels (36). A similar cross-sectional study that examined the effects of the Oxidative Balance Score (OBS) on waist circumference yielded results consistent with ours (37). In a Spanish cohort, DTAC was linked to a lower fasting glucose level among young adults (38). Another study has demonstrated a negative association between CDAI and diabetes as well as hypertension (18, 38). However, we did not observe a direct relationship between CDAI and elevated blood pressure. This can be attributed to the fact that oxidative stress is merely a mediator in the development of hypertension, with chronic inflammation as the primary cause (39). Antioxidant intake alone may not fully counter chronic inflammation, thus limiting CDAI’s therapeutic efficacy against hypertension. It is important to note that this inference is not absolute, as clinical trials have shown that continuous low-dose melatonin intake has shown promise in improving arterial stiffness. Future investigations should explore a wider range of antioxidants in relation to CircS. Discrepancies in outcomes may stem from differences in sample size, demographics, and regional factors.

The utilization of dietary antioxidants in managing depression clinically has been a widely discussed subject. Leiyong Zhao and team identified a stronger negative correlation between CDAI and depression (40), subgroup analyses further showed that the correlation between CDAI and depression differs among various populations, with a notably strong link in older and obese people. In a significant study with 7,274 NHANES participants, Xiong et al. discovered a linear relationship between CDAI and a lower risk of inadequate sleep. Furthermore, they observed a non-linear association between CDAI and reduced risks of obstructive sleep apnea (OSA) and daytime sleepiness (41). These findings highlight the protective role of antioxidant-rich dietary components and provide clinical support for improving sleep health by increasing dietary antioxidants.

We also analyzed the components of CDAI and their impact on CircS. Remarkably, logistic analysis revealed consistent strong negative associations between the intake of Vitamin A, C, E, carotenoids, and CircS, whereas zinc and selenium showed no such trend. Vitamins play a pivotal protective function against CircS. For example, Vitamin A acts as an indirect antioxidant by regulating genes associated with standard antioxidant responses via its metabolite all-trans-retinoic acid. Vitamin E, comprising plant-synthesized compounds, significantly contributes to physiological functions throughout life, notably by impeding lipid peroxidation processes (42). Notably, vitamin C exhibited a linear negative correlation in our RCS study. Ascorbic acid, the active form of vitamin C, was essential for the proper functioning of the central nervous system (43). Concerning potential mechanisms, the nuclear factor erythroid 2-related factor 2 (Nrf2) is essential in controlling the expression of antioxidant enzymes (44). In mice with Nrf2 deficiency, postprandial glucose and TG levels were significantly elevated, with potential involvement in impaired insulin sensitivity in adipose and liver tissues (45, 46). Previous studies indicated that diets rich in vitamin C could enhance the antioxidant enzyme activity by activating the Nrf2/Keap1 signaling pathway (47). Furthermore, vitamin E could activate multiple signaling pathways and regulate apoptosis through the Nrf2-mediated defense system (48). Collectively, it is plausible that higher CDAI may contribute to the Nrf2 supplementation, ultimately reducing the risk of CircS. Multiple studies have underscored the impact of vitamins and carotenoids in ameliorating insufficient sleep and depression, aligning with our findings that elevated intake of vitamins and carotenoids is linked to a decreased risk of CircS. A study uncovered a negative correlation between dietary vitamin C intake and depression. Chioma J. Ikonte et al. have shown a significant inverse relationship between the occurrence of short sleep and insufficient intake of various micronutrients, such as vitamins A, C, and E. As antioxidants, carotenoids could protect the body from age-related chronic diseases caused by oxidative stress (49). Lower level of serum carotenoid concentration was associated with shorter sleep duration (50). Moreover, Deng et al. observed that *β*-cryptoxanthin and lutein were the top two factors linked to a reduced risk of short sleep duration, individuals with higher carotenoid consumption were less prone to experience insufficient sleep, indicating a potential link between optimal sleep duration and increased carotenoid intake (51). Lower vitamin A intake levels were proved to be associated with disturbed wake–sleep cycles, affecting biological rhythms. Presumably, vitamin A and its derivatives could bind to Retinoic acid receptors (RARs) and retinoid X receptors (RXRs), so as to inhibit E-box-dependent clock gene expression mediated by the BMAL1/CLOCK dimer, which is the core circadian rhythm regulation pathway in the suprachiasmatic nucleus (SCN, the central clock) (52–54). Retinoid-related orphan receptor (ROR) served as a crucial link connecting vitamin A and played significant role in regulating the SCN rhythm, it was envolved as a critical part of the molecular mechanism that controls biorhythms and various diseases, including METS, CVD, cancers, and inflammations (55–57). Further evidence on the effects of vitamin A and other antioxidants on circadian rhythms and their underlying mechanisms is warranted.

Numerous studies have explored the correlation between zinc and health outcomes, yet conclusive findings remain elusive. Some studies have reported a notable decrease in serum FBG, HbA1c, TC, LDL-c, and TG levels linked to zinc supplementation (58–61); whereas the detrimental impact of zinc on MetS has been documented by a positive association of zinc status with MetS risk and unfavorable change of blood pressure, glucose, and lipid profile (62–65). For the first time, our study revealed a non-linear U-shaped dose–response association with zinc intake, featuring an inflection point at 12.63 (*p* for nonlinearity <0.001). The dual effects of Zinc could act as both beneficial and harmful to human health, since an optimal range of zinc consumption could lower disease risk, but excessive zinc posed as a risk factor. Indeed, dietary zinc could display both antioxidant and pro-oxidant properties (62). If its concentrations deviate from specific ranges, it could potentially induce the oxidative stress and diminishing antioxidant capacity (66). The physiological mechanisms involved were highly intricate. Matsui and colleagues showed that zinc could be toxic even at micromolar concentrations under certain oxidative stress conditions. They suggested that intracellular zinc ions play a role in the cytotoxicity induced by H_2_O_2_ through two possible mechanisms: (1) H_2_O_2_ increased the permeability of the cell membrane to Zn^2+^, leading to elevated intracellular Zn^2+^ levels following the addition of external ZnCl_2_; (2) H_2_O_2_ reduced cellular non-protein thiol levels, potentially liberating Zn^2+^ from thiol binding sites in the cell (67). Animal studies suggested that prolonged excessive zinc intake from diet might lead to hyperglycemia (68). In rats, excessive zinc intake induced hypertension by activating the renin-angiotensin system (65). However, in our study, both logistic regression and RCS analysis did not reveal a relationship between selenium and CircS (*p* > 0.05). Previous study has also indicated that the antioxidant and pro-oxidant properties of selenium are complex, and no definitive conclusions have been drawn (63, 69). The recommendations regarding dietary supplement dosage of zinc intake should be given with caution, and additional cohort studies are warranted to validate its protective effects on CircS.

Subgroup analysis using logistic regression unveiled an interaction effect between CDAI levels and BMI concerning CircS risk, indicating that BMI could modify the relationship between CDAI and CircS. logistic regression analysis showed that higher CDAI levels were advantageous for individuals without obesity. Conversely, this relationship was not evident in obese individuals. However, additional RCS subgroup analysis indicated that in non-obese participants, increased CDAI levels correlated with reduced CircS risk, with this protective effect reaching a plateau. In contrast, obese individuals exhibited a U-shaped nonlinear association between CDAI and CircS. Prior to the inflection point (1.55), the risk of CircS decreased as CDAI increased; however, beyond this threshold, elevated CDAI levels were linked to an increased disease risk. Increased zinc intake was associated with higher CDAI scores. Obesity is a risk factor for various underlying conditions and has close connections with all components of CircS. Excessive zinc intake may contribute to obesity (60, 62, 68). Therefore, for obese individuals, higher CDAI scores may indicate increased zinc intake, as discussed earlier regarding the dual nature of zinc. Consequently, higher zinc intake could alter the protective effects of vitamins and carotenoids, further exacerbating obesity and increasing the risk of CircS. Thus, elevated CDAI levels may be detrimental for obese individuals.

Our study has notable strengths. Firstly, we used a comprehensive and nationally representative sample of the US population, enhances the reliability of our findings. Secondly, the CDAI, a novel metric, offers a unique approach to evaluating dietary antioxidant intake by considering key antioxidants. Additionally, the adjustment for potential confounding factors enhances the validity of the observed associations. However, it is important to recognize the study’s limitations. Firstly, dietary information was collected only at baseline with no ongoing monitoring, potentially not reflecting daily dietary variations. Secondly, reliance on self-reported 24-h dietary recalls can introduce recall bias or misclassification of dietary intake. Individuals may not accurately recall or report their actual food consumption, potentially introducing inaccuracies in the assessment of the CDAI. However, we observed that fruits and vegetables are the primary dietary sources of vitamin C, and their consumption correlates positively with plasma antioxidants. As a result, serum vitamin levels may more accurately represent our antioxidant intake, thereby enhancing the validation and feasibility of our findings. Moreover, we did not account for distinctions between daily dietary intakes of vitamins or minerals and supplementary intake (e.g., from medications) of these nutrients, could potentially confound the relations between dietary antioxidant intake and Circ syndrome. Lastly, as cross-sectional studies have inherent limitations in establishing causality, adding caution regarding reverse causality in interpretations is essential, further prospective studies are needed.

## 5 Conclusion

In conclusion, this study indicated that a higher CDAI score was correlated with a decreased risk of CircS and its components, with depression and short duration sleep being more prominent. A U-shape correlation was found between Zinc and CircS. Excessively high CDAI levels may be detrimental for obesity. These findings implicated that exploring the protective effects of the combined intake of antioxidants could be a promising avenue for future clinical application.

## Data Availability

Publicly available datasets were analyzed in this study. This data can be found at: https://wwwn.cdc.gov/nchs/nhanes/.

## Footnotes

^1^ https://www.cdc.gov/nchs/nhanes/index.html

^2^version 4.2.3, http://www.R-project.org, The R Foundation
