# Supplementary material for: Association of composite dietary antioxidant index with circadian syndrome: evidence from NHANES
Source: Front Nutr. 2025 Jan 3;11:1501352. doi: 10.3389/fnut.2024.1501352 (PMC11740098; doi:10.3389/fnut.2024.1501352)
Supplement: Supplementary file 1 [file Data_Sheet_1.pdf]

Supplementary Material

1. Supplementary Tables

Table S1. The baseline characteristics grouped by CDAI quantiles.

Characteristic	Total<br>n=11048	CDAI-Q1<br>n=2763	CDAI-Q2<br>n=2761	CDAI-Q3<br>n=2762	CDAI-Q4<br>n=2762	P value
Energy intake, kcal	4206.59 (20.34)	2889.54 (26.76)	3818.01 (31.24)	4462.95 (31.32)	5344.06 (43.18)	< 0.001
Cholesterol intake, mg	286.50(2.38)	181.61(2.84)	254.61(3.31)	312.91(4.92)	372.15(6.17)	< 0.001
Age group, years						0.162
<65	3522 (35.25)	846 (35.86)	846 (35.19)	875 (34.92)	955 (35.15)	
≥65	3791 (38.63)	882 (36.95)	908 (37.15)	999 (39.00)	1002 (40.94)	
Sex	3735 (26.11)	1035 (27.18)	1007 (27.66)	888 (26.09)	805 (23.91)	
Male						< 0.001
Female	5400 (48.40)	1249 (42.74)	1368 (49.51)	1407 (49.81)	1376 (50.51)	
Race	5648 (51.60)	1514 (57.26)	1393 (50.49)	1355 (50.19)	1386 (49.49)	
Other						< 0.001
Non-Hispanic Black	3703 (18.78)	913 (19.80)	934 (18.54)	932 (18.69)	924 (18.29)	
Non-Hispanic White	2166 (10.37)	663 (14.15)	556 (10.92)	490 (9.16)	457 (8.05)	
Poverty income ratio						< 0.001
<1	2072 (12.42)	693 (18.63)	513 (12.35)	447 (10.13)	419 (9.80)	
≥1	8976 (87.58)	2070 (81.37)	2248 (87.65)	2315 (89.87)	2343(90.20)	
Educational attainment						< 0.001
< High school	8674 (85.92)	1918 (77.97)	2150 (86.64)	2259 (87.20)	2347 (90.32)	

≥ High school	2374 (14.08)	845 (22.03)	611 (13.36)	503 (12.80)	415 (9.68)	
BMI						0.011
<30	6782 (62.61)	1666 (61.32)	1633 (59.96)	1716 (63.52)	1767 (65.10)	
≥30	4266 (37.39)	1097 (38.68)	1128 (40.04)	1046 (36.48)	995 (34.90)	
Smoke status						< 0.001
Never	6097 (55.04)	1372 (47.73)	1526(54.93)	1545 (55.92)	1654 (60.08)	
Former	2815 (25.91)	659 (22.68)	714 (26.01)	717 (26.93)	725 (27.37)	
Now	2136 (19.05)	732 (29.59)	521 (19.06)	500 (17.15)	383 (12.55)	
Alcohol consumption						< 0.001
No	1407 (10.19)	443 (12.73)	350 (10.72)	315 (9.31)	299 (8.55)	
Yes	9641 (89.81)	2320 (87.27)	2411 (89.28)	2447 (90.69)	2463 (91.45)	
CircS	2733 (22.13)	798 (25.78)	705 (22.53)	617 (20.50)	613 (20.47)	< 0.001
Elevated glucose	5634 (46.92)	1478 (48.15)	1468 (49.24)	1350 (44.58)	1338 (46.13)	0.045
Elevated HDL-C	3043 (26.50)	869 (30.18)	770 (27.02)	716 (26.06)	688 (23.57)	0.002
Elevated TG	2872 (26.17)	758 (27.78)	730 (27.02)	694 (25.60)	690 (24.70)	0.214
Elevated WC	6398 (57.35)	1670 (59.58)	1679 (60.13)	1577 (57.04)	1472 (53.42)	0.002
Elevated BP	4272 (33.94)	1139 (35.47)	1120 (35.27)	1025 (32.71)	988 (32.73)	0.176
Short sleep duration	1533 (11.59)	484 (16.10)	384 (11.55)	349 (10.00)	316 (9.62)	< 0.001
Depression symptoms	2547 (21.28)	783 (28.38)	594 (19.27)	576 (19.46)	594 (19.21)	< 0.001

Continuous variables were showed as mean (SE), categorical variables were showed as n (%);

All estimates accounted for complex survey designs, and all percentages were weighted;

n, sample size; SE, standard error; CDAI, composite dietary antioxidant index; Q: quartile; BMI, body mass index; HDL-C, High density lipoprotein-cholesterol; TG, triglycerides; WC, waist circumference; BP, blood pressure;

Q1: CDAI ≤ -2.191; Q2: -2.191 < CDAI ≤ 0.044; Q3: 0.046 <CDAI ≤ 2.765; Q4: CDAI > 2.765.

Table S2. Sample characteristics by MetS and CircS status

Groups according to the MetS definition

	Total	Normal	MetS
	n=11048	n=6947	n=4101
Weighted prevalence (%)	100	65.72	34.28

Groups according to the CircS definition

	Total	Normal	CircS alone	MetS alone	MetS and CircS
	n=11048	n=6801	n=146	n=1514	n=2587
Weighted prevalence (%)	100	64.63	1.09	13.24	21.04

Table S3 Association of Composite dietary antioxidant index with circadian syndrome components.

	Crude model		Model 1		Model 2	
	OR 95%CI	P	OR 95%CI	P	OR 95%CI	P
**Elevated glucose**						
Q1	1 [Reference]		1 [Reference]		1 [Reference]	
Q2	1.04 (0.90,1.21)	0.561	0.99 (0.84,1.18)	0.936	0.97 (0.82,1.14)	0.687
Q3	0.87 (0.75,1.00)	0.052	0.80 (0.67,0.95)	0.012	0.76 (0.64,0.90)	0.001
Q4	0.92 (0.80,1.07)	0.268	0.85 (0.69,1.05)	0.135	0.80 (0.65,0.98)	0.031
P for trend		0.068		0.046		0.007
Per 1-SD increase	0.97 (0.91,1.03)	0.355	0.94 (0.85,1.04)	0.239	0.93 (0.82,1.04)	0.213
**Elevated TG**						
Q1	1 [Reference]		1 [Reference]		1 [Reference]	
Q2	0.96 (0.82,1.13)	0.644	0.88(0.74,1.04)	0.135	0.90 (0.76,1.08)	0.263
Q3	0.89 (0.76,1.05)	0.165	0.78(0.65,0.93)	0.008	0.80 (0.66,0.97)	0.027
Q4	0.85 (0.73,1.00)	0.054	0.70(0.57,0.86)	<0.001	0.74 (0.59,0.92)	0.006
P for trend		0.037		<0.001		0.005
Per 1-SD increase	0.92 (0.86,0.97)	0.004	0.83 (0.76,0.91)	<0.001	0.84 (0.77,0.92)	<0.001
**Elevated BP**						
Q1	1 [Reference]		1 [Reference]		1 [Reference]	
Q2	0.99 (0.84,1.16)	0.912	0.98 (0.79,1.21)	0.853	0.96 (0.78,1.19)	0.721
Q3	0.88 (0.76,1.03)	0.105	0.87 (0.72,1.05)	0.151	0.84 (0.70,1.02)	0.081
Q4	0.89 (0.77,1.02)	0.100	0.90 (0.71,1.14)	0.400	0.86 (0.68,1.10)	0.223
P for trend		0.031		0.230		0.108
Per 1-SD increase	0.95 (0.91,0.99)	0.021	0.94 (0.87,1.00)	0.049	0.92 (0.86,0.99)	0.033

**Elevated WC**						
Q1	1 [Reference]		1 [Reference]		1 [Reference]	
Q2	1.02 (0.90,1.16)	0.727	1.05 (0.91,1.22)	0.484	0.99 (0.85,1.15)	0.861
Q3	0.90 (0.77,1.05)	0.184	0.90 (0.75,1.07)	0.226	0.81 (0.68,0.97)	0.022
Q4	0.78 (0.67,0.91)	0.002	0.75 (0.60,0.92)	0.008	0.65 (0.52,0.80)	<0.001
P for trend		<0.001		0.002		<0.001
Per 1-SD increase	0.88 (0.83,0.93)	<0.000	0.84 (0.77,0.91)	<0.001	0.79 (0.73,0.87)	<0.001
**Reduced HDL-C**						
Q1	1 [Reference]		1 [Reference]		1 [Reference]	
Q2	0.86 (0.73,1.00)	0.056	0.88 (0.75,1.04)	0.133	0.92 (0.78,1.08)	0.321
Q3	0.82 (0.68,0.97)	0.025	0.82 (0.68,0.98)	0.030	0.86 (0.71,1.03)	0.108
Q4	0.71 (0.60,0.85)	<0.001	0.69 (0.56,0.85)	<0.001	0.75 (0.61,0.92)	0.008
P for trend		<0.001		<0.001		0.006
Per 1-SD increase	0.86 (0.80,0.91)	<0.001	0.82 (0.75,0.90)	<0.001	0.84 (0.77,0.92)	<0.001
**Depression**						
Q1	1 [Reference]		1 [Reference]		1 [Reference]	
Q2	0.60 (0.50,0.72)	<0.001	0.59 (0.49,0.71)	<0.0001	0.64 (0.53,0.78)	<0.001
Q3	0.61 (0.51,0.73)	<0.001	0.56 (0.45,0.69)	<0.0001	0.62 (0.50,0.77)	<0.001
Q4	0.60 (0.52,0.70)	<0.001	0.49 (0.40,0.61)	<0.0001	0.59 (0.48,0.72)	<0.001
P for trend		<0.001		<0.001		<0.001
Per 1-SD increase	0.85 (0.80,0.91)	<0.001	0.78 (0.71,0.85)	<0.001	0.84 (0.77,0.92)	<0.001
**Short sleep duration**						
Q1	1 [Reference]		1 [Reference]		1 [Reference]	
Q2	0.68 (0.58,0.80)	<0.001	0.65 (0.55,0.78)	<0.001	0.70 (0.59,0.83)	<0.001

Q3	0.58 (0.48,0.71)	<0.001	0.53 (0.42,0.67)	<0.001	0.58 (0.46,0.73)	<0.001
Q4	0.56 (0.45,0.68)	<0.001	0.46 (0.35,0.61)	<0.001	0.54 (0.41,0.70)	<0.001
P for trend		<0.001		<0.001		<0.001
Per 1-SD increase	0.83 (0.76,0.90)	<0.001	0.77 (0.69,0.87)	<0.001	0.82 (0.74,0.92)	<0.001

Crude model: Unadjusted model.

Model 1: Adjusted for age, sex, race, PIR, educational level and energy intake.

Model 2: Additionally adjusted for cholesterol intake, smoke status, and alcohol consumption.

PIR, poverty income ratio; TG, triglycerides; BP, blood pressure; WC, waist circumference; HDL-C, High density lipoprotein-cholesterol; SD, standard deviation.

Table S4 Association of Composite dietary antioxidant index components with circadian syndrome

	Crude model		Model 1		Model 2	
	OR 95%CI	P	OR 95%CI	P	OR 95%CI	P
**Vitamin A**						
Q1	1 [Reference]		1 [Reference]		1 [Reference]	
Q2	0.98 (0.82,1.17)	0.809	0.86 (0.71,1.05)	0.134	0.88 (0.72,1.07)	0.194
Q3	0.83 (0.70,0.98)	0.032	0.68 (0.57,0.82)	<0.001	0.70 (0.58,0.84)	<0.001
Q4	0.79 (0.65,0.95)	0.014	0.63 (0.50,0.78)	<0.001	0.64 (0.51,0.81)	<0.001
P for trend		0.003		<0.001		<0.001
**Vitamin C**						
Q1	1 [Reference]		1 [Reference]		1 [Reference]	
Q2	0.86 (0.74,1.02)	0.077	0.80 (0.68,0.95)	0.009	0.83 (0.70,0.98)	0.032
Q3	0.89 (0.76,1.06)	0.196	0.79 (0.65,0.95)	0.014	0.82 (0.69,1.00)	0.050
Q4	0.74 (0.63,0.88)	< 0.001	0.64 (0.53,0.78)	<0.001	0.69 (0.57,0.85)	< 0.001
P for trend		0.002		<0.001		< 0.001
**Vitamin E**						
Q1	1 [Reference]		1 [Reference]		1 [Reference]	
Q2	0.80 (0.68,0.94)	0.008	0.78 (0.65,0.93)	0.007	0.80 (0.67,0.96)	0.016
Q3	0.75 (0.62,0.90)	0.003	0.72 (0.58,0.89)	0.003	0.74 (0.60,0.92)	0.007
Q4	0.65 (0.55,0.78)	<0.001	0.60 (0.48,0.76)	<0.001	0.64 (0.51,0.81)	<0.001
P for trend		<0.001		<0.001		<0.001
**Zinc**						
Q1	1 [Reference]		1 [Reference]		1 [Reference]	
Q2	0.80 (0.67,0.95)	0.011	0.83 (0.69,1.00)	0.047	0.84 (0.70,1.01)	0.068

Q3	0.77 (0.66,0.90)	0.001	0.86 (0.72,1.02)	0.085	0.87 (0.73,1.05)	0.139
Q4	0.79 (0.67,0.93)	0.005	0.96 (0.77,1.21)	0.735	0.98 (0.78,1.24)	0.895
P for trend		0.005		0.776		0.936
**Selenium**						
Q1	1 [Reference]		1 [Reference]		1 [Reference]	
Q2	0.86 (0.74,1.00)	0.053	0.94 (0.80,1.10)	0.406	0.94 (0.80,1.10)	0.425
Q3	0.79 (0.67,0.93)	0.005	0.98 (0.82,1.17)	0.822	0.97 (0.81,1.17)	0.764
Q4	0.80 (0.69,0.93)	0.004	1.15 (0.93,1.41)	0.187	1.11 (0.89,1.38)	0.369
P for trend		0.005		0.219		0.425
**Carotenoid**						
Q1	1 [Reference]		1 [Reference]		1 [Reference]	
Q2	0.85 (0.71,1.01)	0.060	0.88 (0.73,1.06)	0.174	0.90 (0.75,1.08)	0.246
Q3	0.77 (0.64,0.92)	0.004	0.83 (0.69,1.02)	0.071	0.86 (0.70,1.05)	0.134
Q4	0.78 (0.67,0.91)	0.002	0.85 (0.70,1.02)	0.079	0.88 (0.73,1.07)	0.189
P for trend		0.001		0.074		0.182

Crude model: Unadjusted model.

Model 1: Adjusted for age, sex, race, PIR, educational level and energy intake.

Model 2: Additionally adjusted for cholesterol intake, smoke status, and alcohol consumption.

Table S5. Sensitivity analysis of the association between CDAI and CircS using unweighted logistic regression

	Crude model		Model 1		Model 2	
	OR 95%CI	***P***	OR 95%CI	***P***	OR 95%CI	***P***
**Circs**						
Q1	1 [Reference]		1 [Reference]		1 [Reference]	
Q2	0.86 (0.79,0.93)	<0.001	0.87 (0.80,0.95)	0.002	0.89 (0.81,0.97)	0.008
Q3	0.73 (0.67,0.79)	<0.001	0.76 (0.69,0.83)	<0.0001	0.77 (0.70,0.85)	<0.001
Q4	0.64 (0.59,0.70)	<0.001	0.67 (0.60,0.74)	<0.0001	0.68 (0.61,0.77)	<0.001
P for trend		<0.001		<0.001		<0.001

Crude model: Unadjusted model.

Model 1: Adjusted for age, sex, race, PIR status, educational level and energy intake.

Model 2: Additionally adjusted for cholesterol intake, smoke status, alcohol consumption.

CDAI, composite dietary antioxidant index; CircS, circadian syndrome; PIR, poverty income ratio.

Table S6. Sensitivity analysis of association between CDAI and CircS after additionally adjusting for physical activity

	Crude model		Model 1		Model 2	
	OR 95%CI	***P***	OR 95%CI	***P***	OR 95%CI	***P***
**Circs**						
Q1	1 [Reference]		1 [Reference]		1 [Reference]	
Q2	0.74 (0.56,0.99)	0.042	0.71 (0.52,0.97)	0.033	0.71 (0.52,0.97)	0.029
Q3	0.73 (0.52,1.01)	0.055	0.68 (0.47,0.97)	0.036	0.69 (0.48,0.99)	0.045
Q4	0.79 (0.60,1.03)	0.086	0.70 (0.47,1.03)	0.067	0.76 (0.52,1.11)	0.155
P for trend		0.133		0.101		0.227

Crude model: Unadjusted model.
Model 1: Adjusted for age, sex, race, PIR status, educational level and energy intake.
Model 2: Additionally adjusted for cholesterol intake, smoke status, alcohol consumption, and physical activity.

CDAI, composite dietary antioxidant index; CircS, circadian syndrome; PIR, poverty income ratio.

2. Supplementary Figure

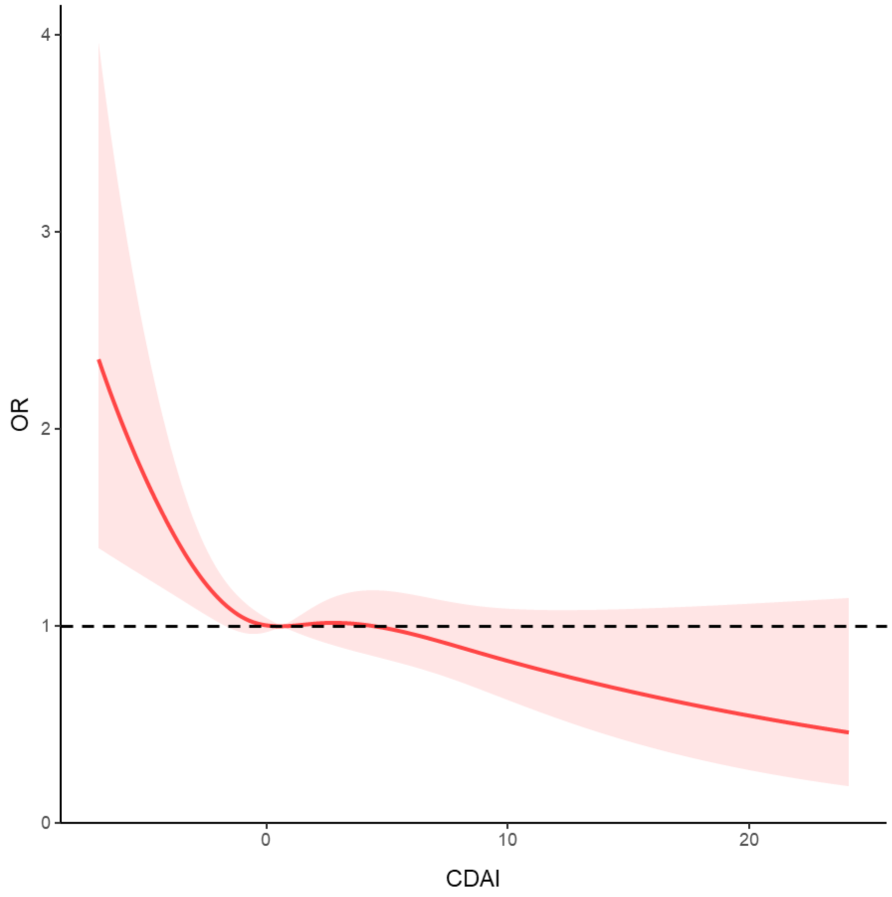

Figure S1. The RCS curve of the association between CDAI and CircS after additionally adjusting for physical activity. Adjusted for age, sex, race, poverty income ratio, educational level, energy intake, cholesterol intake, smoke status, alcohol consumption, and physical activity.
